# Strong Dietary Restrictions Protect *Drosophila* against Anoxia/Reoxygenation Injuries

**DOI:** 10.1371/journal.pone.0005422

**Published:** 2009-05-01

**Authors:** Paul Vigne, Michel Tauc, Christian Frelin

**Affiliations:** 1 IBDC, CNRS UMR 6543, Université de Nice Sophia Antipolis, Nice, France; 2 TIANP, CNRS FRE 3093, Université de Nice Sophia Antipolis, Nice, France; UT MD Anderson Cancer Center, United States of America

## Abstract

**Background:**

Reoxygenation of ischemic tissues is a major factor that determines the severity of cardiovascular diseases. This paper describes the consequences of anoxia/reoxygenation (A/R) stresses on *Drosophila*, a useful, anoxia tolerant, model organism.

**Methodology/Principal Findings:**

Newly emerged adult male flies were exposed to anoxic conditions (<1% O_2_) for 1 to 6 hours, reoxygenated and their survival was monitored.

**Results:**

A/R stresses induced a transient increase in mortality which peaked at the time of reoxygenation. Then flies recovered low mortality rates similar to those of control flies. A/R induced mortality was strongly dependent on dietary conditions during the 48 h that preceded anoxia. Well fed flies were anoxia sensitive. Strong dietary restrictions and starvation conditions protected flies against A/R injuries. The tolerance to anoxia was associated to large decreases in glycogen, protein, and ATP contents. During anoxia, anoxia tolerant flies produced more lactate, less phosphate and they maintained more stable ATP levels than anoxia sensitive flies. Moderate dietary restrictions, which increased the longevity of normoxic flies, did not promote resistance to A/R stresses. Diet dependent A/R injuries were still observed in sima loss of function mutants and they were insensitive to dietary rapamycin or resveratrol. AICAR (5-aminoimidazole-4-carboxamide-1-beta-D-ribosefuranoside), an activator AMP kinase decreased A/R injuries. Mutants in the insulin signalling pathway were more anoxia tolerant in a fed state.

**Conclusion/Significance:**

Long A/R stresses induce a transient increase in mortality in *Drosophila*. This mortality is highly dependent on dietary conditions prior to the stress. Strong dietary restrictions and starvation conditions protect flies against A/R injuries, probably by inducing a major remodelling of energy metabolism. The results also indicate that mechanistically different responses develop in response to dietary restrictions of different strengths. AMP kinase and the insulin signalling pathway are possible mediators of diet dependent anoxic tolerance in *Drosophila*.

## Introduction

The ability of organisms to sustain O_2_ deprivation is highly variable. Human brain, cardiac and renal tissues are highly vulnerable to hypoxia and irreversible injuries occur within a few minutes of blood flow arrest. Some animal species are much more tolerant to oxygen deprivations. Sperm whales and seals may dive to more than 1000 m and remain submerged for 2 hours. Some turtles survive without oxygen for up to four months. The susceptibility or tolerance to O_2_ deprivation involves complex cellular and systems level adaptations that have only recently been considered [1], [2]. They are of major interest. Innovative pharmacological strategies are eagerly needed to increase the tolerance of human ischemic tissues to the absence of oxygen.

There are different forms of hypoxic/anoxic stresses and each of them is associated to specific diseased states in humans. Chronic hypoxic conditions are encountered in a few pathological situations such as pulmonary hypertension. Acute anoxia is a consequence of blood flow arrest and is associated to major cardiovascular diseases such as stroke and myocardial infarction. Anoxic/hypoxic episodes are often transient as tissues can be reperfused and reoxygenated, for instance following angioplasty procedures. Reperfusion of hypoxic/anoxic tissues induces a massive production of reactive oxygen species [3] and a major reorganization of ion fluxes across the plasma membrane of excitable cells [4]. The two mechanisms contribute to cell death.

*Drosophila melanogaster* is increasingly used as a model organism for cardiac and neurological diseases [5], [6]. It is an obvious candidate organism for assessing the mechanisms involved in the sensitivity of a whole organism to hypoxia/anoxia and for identifying new molecular targets that might lead to the development of innovative therapeutic strategies. Flies are much more tolerant to hypoxia/anoxia than humans [7], [8]. We previously analysed the responses of flies to chronic hypoxic conditions (5% O_2_) and reported that feeding flies on a protein diet reduces their longevity under chronic hypoxic conditions [9]. The effect of dietary proteins is mimicked by individual amino acids and by polyamines. It is reduced by inhibitors of polyamine synthesis and of eIF5A hypusination [10]. This study concerns the responses of flies to anoxic (<1% O_2_) conditions. We report here that strong dietary restrictions protect flies against anoxia/reoxygenation stresses probably by inducing a unique, insulin and AMP kinase dependent, hypometabolic state.

## Results

### Demographic analysis consequences of A/R stresses

Flies responded to acute anoxia (<1% O_2_) by a stereotyped response that had previously been described [11]. Briefly, flies became uncoordinated within 1 minute and fell down to the bottom of the tubes. They rapidly stopped moving and stood motionless. Following reoxygenation, flies woke up after some delay and resumed normal activities. Mortality was negligible if the anoxic period was <1 h. Anoxic periods >1 h killed flies however. It is important to note that the effect of anoxia cannot be dissociated from that of reoxygenation. Flies have to be reoxygenated in order to assess their survival. As a consequence, anoxia followed by reoxygenation will be referred to as an anoxia/reoxygenation stress (A/R stress)

**Figure 1:**
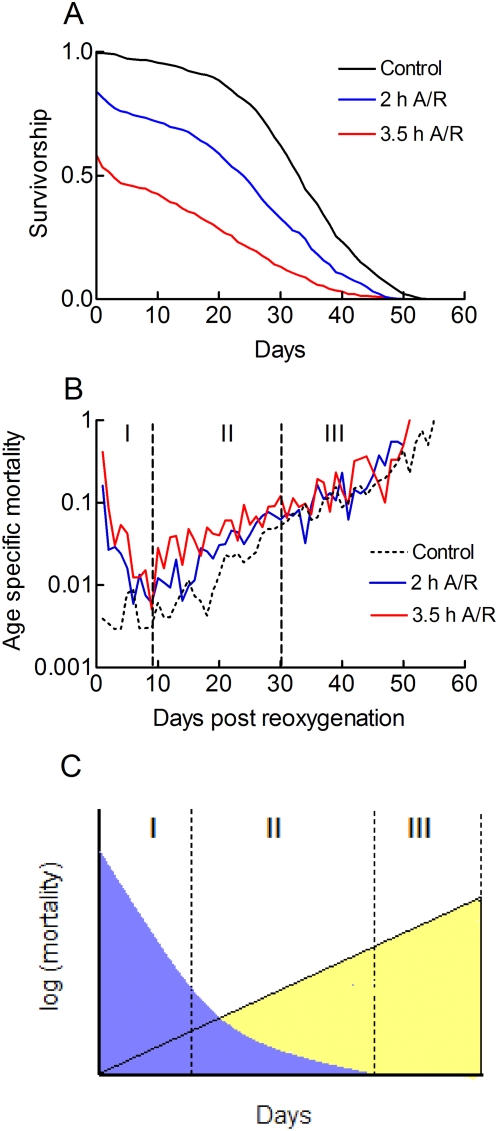
Long A/R stresses induce a transient increase in mortality rates. A. Survivorship of A/R stressed flies. Male w^1118^ flies were exposed to 2 h or 3.5 h of anoxia (∼1% O_2_), reoxygenated and survivorships curves were determined. Demographic parameters were: median longevity (control, 32 days, 2 h A/R stress, 26 days, 3.5 h A/R stress, 3.5 days), maximum longevity (control, 50 days, 2 h A/R stress, 46 days, 3.5 h A/R stress, 38 days), sample sizes (control, 1021, 2 h A/R stress, 886, 3.5 h A/R stress, 862). B. Age specific mortalities were plotted against age using a log scale. The trajectory mortality of control flies (dotted black) is consistent with a Gompertz model. Three phases, labelled I, II and III, are distinguished (see text). A Cox regression analysis was performed. A 2 h A/R stress increased the relative risk of death 8.6 fold (95%CI: 6.0–12.3). A 3.5 h A/R stress increased the relative risk of death 20.1 fold (95% CI: 14.2–28.5). Sample sizes were: control, 1021, 2 h A/R stress, 886, 3.5 h A/R stress, 862. After 10 days of recovery, relative risks of death were 2.19 (95% CI: 1.85–2.60) and 1.79 (95% CI: 1.63–1.96) for 2 h A/R stressed flies and 3.5 h A/R stressed flies respectively. Sample sizes were: control, 987, 2 h A/R stress, 655, 3.5 h A/R stress, 388. After 30 days of recovery, relative risks of death were: 1.26 (95% CI: 1.11–1.44) and 1.22 (95% CI: 1.12–1.34 for 2 h A/R stressed flies and 3.5 h A/R stressed flies respectively. Sample sizes were: control, 725, 2 h A/R stress, 353, 3.5 h A/R stress, 159. C. Interpretation of the data. Two mortality components are defined. (i) An A/R stress induced mortality that reached a maximum 24 h after reoxygenation and that declines (blue) and (ii) the age associated exponential increase in mortality rate (yellow).

**Figure 2:**
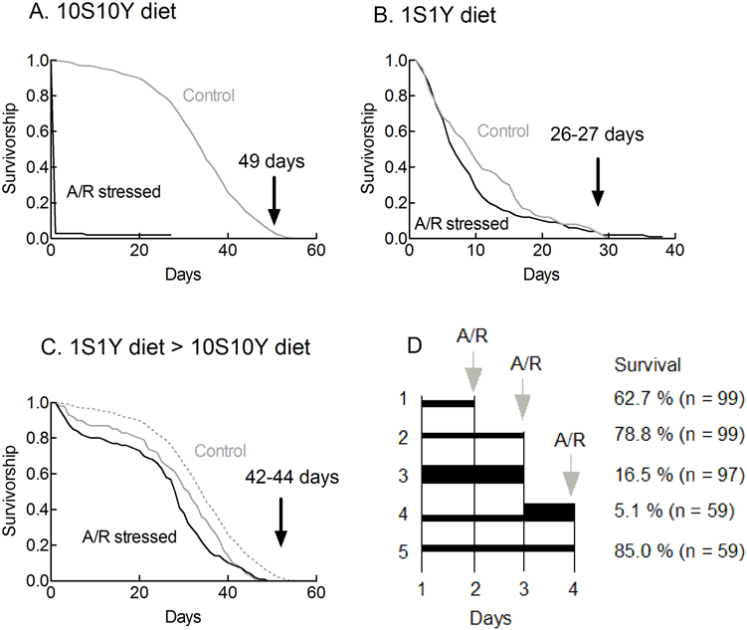
A severe dietary restriction protects flies against A/R injuries. A. Fed flies were anoxia sensitive. One day old flies were fed on a rich 10S10Y diet. After 2 days, flies were exposed to 3.5 hours of severe anoxia (<0.1% O_2_) and reoxygenated. The survivorship curve (black) is compared to that of normoxic flies on the same diet (grey). Few flies (∼3%) recovered from the A/R stress. The largest mortality as compared to the experiments shown in Figure 1 was due to the use of more severe anoxic conditions (0.1% O_2_ versus 1% O_2_). Sample sizes were 109 (controls) and 120 (A/R stressed flies). B. Dietary restricted flies were anoxia resistant. One day old flies were fed on a poor 1S1Y diet. After 2 days, flies were exposed to 3.5 hours of anoxia, reoxygenated and maintained on the same 1S1Y diet. The survivorship curve (black) is compared to that of normoxic flies maintained on a 1S1Y diet (grey). P = 0.0095 using the Log rank test. Mean longevities were 10.2±0.77 days (n = 98) and 8.43±0.50 days (n = 219) for control and A/R stressed flies respectively. C. An assay for the diet dependent tolerance to A/R stresses. Survivorship curves of three groups of flies are compared. Grey continuous curve: One day old flies were fed on a 1S1Y diet and switched after 2 days to a 10S10Y diet. Black curve. One day old flies were fed for 2 days on a 1S1Y diet, A/R stressed (3.5 h) and switched to a 10S10Y diet. The difference between the two curves documents the influence of the A/R stress on dietary restricted flies. An A/R stress mainly increased the short term mortality. The dotted grey line shows for comparison the survivorship curve of normoxic flies maintained on a 10S10Y diet. The difference with the continuous grey line documents the action of a 1S1Y to 10S10Y diet shift on longevity. The comparison shows that shifting flies from a 1S1Y diet to a 10S10Y diet rapidly restored low mortality rates. Arrows in panels A–C show maximum longevities. D. Influence of different protocols of dietary restriction on survival following an A/R stress. One day old flies were maintained on a 1S1Y diet (thin lines) or a 10S10Y diet (thick lines) for the times indicated and then exposed to a 3.5 h A/R stress. Surviving flies were scored after 48 hours. Five different experimental protocols are compared. Experiments #2 and #3 reproduced the data presented in panels A and C. Experiment #1 shows that a one day adaptation to a 1S1Y diet did not induce a maximum protection against A/R stresses. Experiment #4 shows that the protective action of a 1S1Y diet was completely lost when flies were shifted back to a 10S10Y diet for 24 hours.

We first analysed the demography of large cohorts of flies which were exposed to long periods of anoxia and reoxygenated. Figure 1A compares complete survivorship curves. It shows that 2 to 3.5 h A/R stresses reduced the short term survival of the flies and decreased their maximum longevity. Figure 1B analyses age specific mortalities. It shows that mortality rates increased exponentially with age in the control fly population. This relationship was expected from the Gompertz model. Figure 1B further shows that A/R stresses induced rapid and transient increases in mortality which peaked soon after reoxygenation. Then, mortality rates decreased. Three steps could be distinguished. During phase I (1–10 days), mortality rates decreased with time and they remained much larger than for controls flies that had not been A/R stressed. During phase II (10–30 days), mortality rates increased in the three groups of flies and the difference between A/R stressed flies and controls decreased. During phase III (>30 days), mortality rates of control and A/R stressed flies were similar and they all increased with age. A demographic analysis using the Cox model was performed. Results showed that a 3.5 h A/R stress increased the relative risk of death 20 fold. A 1.8 fold increase in the risk of death was still observed after 10 days of recovery. Similarly, a 2 h A/R stress increased the relative risk of death 8.6 fold. A 2.2 fold increase was still observed after 10 days. Mortality rates in control and A/R stressed flies were similar after 30 days. Figure 2C presents an interpretation of the data. It considers the age specific mortality as the sum of two independent mortality components: (i) An A/R stress induced mortality that was greatest at the time of reoxygenation and that declined steadily with age. (ii) A mortality component which was associated to normal ageing and whose rate increased exponentially with age.

A/R induced injuries in mammals are well known to result from the large oxidative burst that accompanies the reoxygenation of the tissues [3]. The possible contribution of reactive oxygen species to A/R induced injuries in flies was assessed using an exogenous antioxydant molecule. Euk-8 is a superoxide dismutase/catalase mimetic which partially rescues the phenotype of superoxide dismutase deficient flies [12]. Feeding flies on a Euk-8 diet reduced the mortality induced by a 3.5 h A/R stress (measured 48 hours after reoxygenation) from 42% to 24% (p<0.01). This indicated that reactive oxygen species contributed to A/R injuries.

### A/R injuries are diet dependent

The sensitivity of flies to chronic hypoxic conditions has previously been shown to be highly dependent on dietary conditions [9], [10], [13]. This study analyses the dietary dependence of A/R induced injuries. Figure 2A shows that flies fed on a rich 10S10Y diet were highly sensitive to A/R stresses. Under the conditions used (<0.1% O_2_), only a few flies woke up after reoxygenation. Figure 2B shows that flies fed on a poor 1S1Y diet had a reduced longevity as compared to flies maintained on a 10S10Y diet (mean longevity: 10 days vs 27 days). Yet, they were largely insensitive to a 3.5 h A/R stress. Most dietary restricted flies woke up after reoxygenation. They then died as a consequence of the poor dietary conditions and possibly as a consequence of the A/R stress. To better define A/R stress induced mortality and assess its diet dependence, we defined a diet switch protocol in which all flies were switched to a rich 10S10Y diet after reoxygenation. Figure 2C shows the results of a control experiment in which normoxic flies were fed on a 1S1Y diet for 2 days and then switched to a 10S10Y diet for the rest of their lives. It shows that flies rapidly recovered low mortality rates after the switch to a 10S10Y diet. This result fully agrees with previous reports [14], [15] which indicated that dietary shifts induced rapid changes in mortality rates. In a second series of experiments, flies were adapted for 2 days to a 1S1Y diet, exposed to a 3.5 h anoxia, reoxygenated and transferred to a 10S10Y diet for the rest of their lives. Figure 2C shows that under these conditions, the A/R stress induced a modest and transient increase in mortality. Data can be summarized as follows. Flies adapted for 2 days to a rich 10S10Y diet did not wake up after the A/R stress. Most flies adapted to a 1S1Y diet woke up after the A/R stress. Surviving flies maintained on a poor diet were short lived (as did flies which were not A/R stressed). Flies switched to a rich diet were long lived. These results indicated that dietary conditions prior to the A/R stress determined the severity of A/R injuries.

Figure 2D further analyses the consequences of different dietary manipulations on survival following a 3.5 h A/R stress. It shows that a one day adaptation to a 1S1Y diet was not sufficient to induce maximum protection. It also shows that protection against A/R stresses was rapidly lost after switching flies to nutrient rich, 10S10Y, conditions. Thus, diet changes induced rapid (24–48 h) and reversible changes in the sensitivity of the flies to A/R stresses. In all subsequent experiments flies were adapted for 2 days to different diets, A/R stressed and switched to a 10S10Y diet. Mortality was assessed after 2 days.

**Figure 3:**
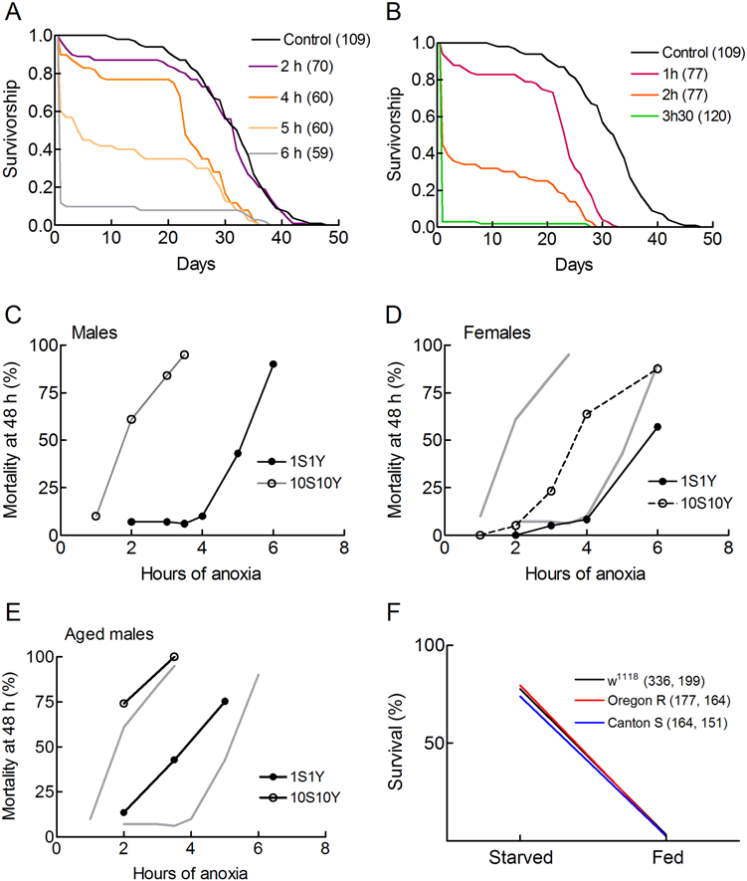
Influence of the duration of anoxia on mortality. A, B. Male w^1118^ flies were adapted for 2 days to a 1S1Y diet (A) or a 10S10Y diet (B), exposed to the indicated times of anoxia, reoxygenated, switched to a 10S10Y diet and survivorship curves were determined. Sample sizes are indicated in parentheses. Note that A/R stresses mainly reduce the short term mortality and have less effect on maximum longevities. C–E. Relationship between the duration of anoxia and the short term mortality for young male flies (C), young female flies (D) and 1 month old male flies (E). In panels D and E, the grey lines reproduce the curves obtained for young males (panel C). Sample sizes were 40–113. Aged males were maintained on a standard food medium for 1 month, switched either to a 1S1Y or a 10S10Y diet for two days and then A/R stressed. F. Norms of reaction of flies of different strains as indicated. Young male flies were used. Sample sizes (starved, fed) are indicated in parentheses. Differences between strains are not statistically significant.

Figures 3A and 3B compare survivorship curves of flies exposed to A/R stresses of different durations. Flies were either adapted to a poor, 1S1Y, diet (Figure 3A) or to a rich, 10S10Y, diet (Figure 3B). Under all conditions, the A/R stress increased the short term mortality and had less consequence on maximum longevity. Figure 3C shows a plot of A/R induced mortality as a function of the duration of the anoxic stress. It shows that young male flies fed on the poor diet were much more anoxia tolerant than well fed flies. A 3.5 h A/R stress killed most fed flies. A 6 h A/R stress was required to kill all flies adapted to the 1S1Y diet. Thus, flies on a poor diet resisted 2.5 h longer anoxia.

Poor diets also protected young female flies against A/R stresses. Fed females were more anoxia tolerant than fed males. As a consequence they were less sensitive to dietary manipulations than males (Figure 3D). The effect of ageing on anoxic tolerance was assessed by comparing young males and aged, 1 month old, males. Figure 3E shows that aged males on a poor diet were less anoxia tolerant than young males on a poor diet. As a consequence, aged males were less sensitive to dietary manipulations.

The responses of *Drosophila* to changes in diet are well known to be highly dependent on genetic background [16]. We therefore analysed how flies of the Canton S and Oregon R strains responded to a 3.5 h A/R stress. Figure 3F shows that w^1118^, Canton S and Oregon R flies had identical sensitivities to A/R stresses and diet dependences.

### Moderate dietary restrictions did not protect against A/R injuries

**Figure 4:**
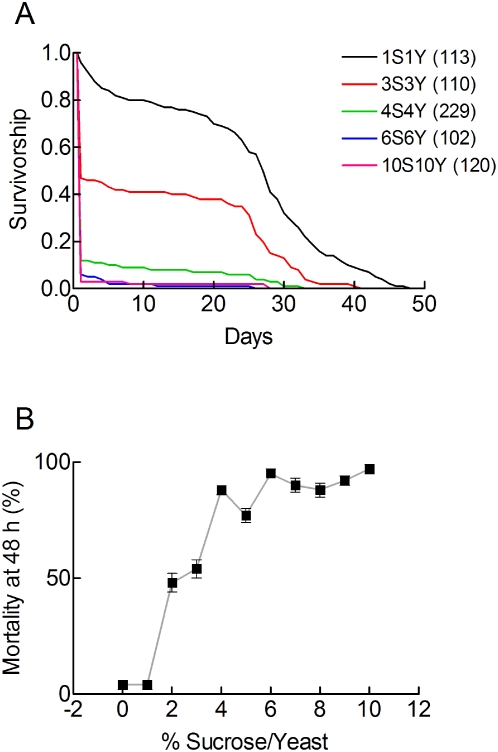
Diet dependent anoxic tolerance. Male w^1118^ flies were adapted for 2 days to nutrient media that consisted of equal amounts of yeast and sucrose, exposed to 3.5 h of anoxia, reoxygenated and switched to a 10S10Y diet for the rest of their lives. A. Survivorship curves. Note that different dietary conditions mainly changed the short term mortality and that the maximum longevity was less affected. Sample sizes are indicated in parentheses. B. Influence of serial dilutions of a 10S10Y nutrient medium on A/R induced mortality. The total number of flies used was 1501. Sample sizes were 103 to 229. Means±sem are indicated.

The previous experiments compared the responses of flies exposed to nutrient rich (10S10Y) or nutrient poor (1S1Y) conditions. We then defined the sensitivity of A/R injuries to dietary restrictions of different strengths. Flies were fed for 2 days on coordinate dilutions of a rich 10S10Y medium, exposed to a 3.5 h A/R stress and their survivorship was analysed. Figure 4A shows that changes in food quality increased the short term mortality. They had less consequence on the maximum longevity. Figure 4B shows that A/R induced mortality was large for rich diets that contained more than 4% of sucrose and 4% yeast. It was intermediate for 2S2Y and 3S3Y diets. It was the lowest for the 1S1Y diet and for wet starvation conditions. Thus, only strong dietary restrictions and wet starvation conditions protected flies against A/R stresses. It is important to note that flies fed on a 1S1Y diet were not starving. Their mean life span under normoxic conditions was 10.5±0.4 days (n = 196), 2.6 times longer than the mean life span of starving flies (4.1±0.1 days, n = 280). In comparison the mean life span of flies maintained on a 10S5Y diet was 78.6 days [15].

Dietary yeast and sucrose have recently been shown to have distinct, non additive actions of the longevity of normoxic or hypoxic flies. Pure yeast diets favour a lean phenotype. Pure sucrose diets favour an obese phenotype [17]. Flies on a pure yeast diet are highly sensitive to chronic hypoxia. Flies on a pure sucrose diet are more hypoxia resistant [13]. We therefore analysed the effects of dietary yeast and sucrose on A/R induced mortality. Flies were exposed either to a pure 10% sucrose or to a pure 10% yeast diet for 2 days and the mortality induced by a 3.5 h A/R stress was measured. It was 94% (n = 120) for yeast adapted flies and 76% (n = 240) for sucrose adapted flies (p<0.01). This difference indicated that sucrose adaptation protected flies against A/R stresses. It should be noted however a starvation stress afforded a much greater protection (<10% mortality, Figure 4B). This indicated that the tolerance to A/R stresses was not dependent on specific nutrients. It was only increased by strong dietary restrictions and starvation conditions.

### Diet dependent remodelling of energy metabolism

An obvious hypothesis for the previous results could be that adaptation to different diets modified the body composition and energetic status of the flies. Flies were maintained for two days either on a 1S1Y diet or a 10S10Y diet to produce anoxia tolerant and anoxia sensitive flies. Flies were then frozen and their body composition was analysed. Table 1 shows that anoxia tolerant flies had decreased ATP (−40%), lactate (−72%), phosphate (−17%), glycogen (−31%), triglycerides (−10%) and protein (−34%) contents.

**Table 1:** Body composition of anoxia tolerant and sensitive flies.

	Anoxia tolerant 21% O_2_	Anoxia sensitive 21% O_2_	Anoxia tolerant 1 h N_2_	Anoxia sensitive 1 h N_2_
ATP (AU/fly)	13,597±3,612 (3)	22,783±369 (3)	7,894±1,089 (3)	10,759±955 (3) §
Lactate (nmoles/fly)	2.39±0.39 (5)	8.60±1.17 (5)	10.83±2.82 (5)	15.23±1.62 (5)
Phosphate (nmoles/fly)	7.58±0.36 (5)	9.20±0.26 (5)	12.39±0.77 (5)	17.48±0.77 (5)
Glycogen (µg/fly)	6.47±0.28 (3)	9.47±0.38 (3)	ND	ND
Triglycerides (AU/fly)	263.0±2.7 (6)	294.0±2.9 (8) §§	ND	ND
Proteins (µg/fly)	47.76±1.85 (8)	57.1±1.48 (9)	ND	ND

Male w^1118^ flies were fed for 2 days either on a 1S1Y diet or a 10S10Y diet to produce anoxia tolerant or anoxia sensitive states. Flies were then killed (columns 2 and 3) or exposed to a 1 h anoxia (columns 4 and 5) and killed. Total levels of ATP, lactate, phosphate, glycogen, triglycerides and proteins were determined as described in Materials and Methods. Means±sem and the number of independent experiments performed are indicated. AU: arbitrary units, ND: Not determined. §: p>0.05 as compared to starvation stressed flies after 1 h of anoxia. §§: p>0.05 as compared to starvation stressed flies. Other differences were statistically significant (p<0.01).

Anoxia is followed by a rapid cessation of oxidative metabolism and the activation of anaerobic glycolysis [18], [19]. We therefore analysed the consequence of a 1 h anoxia on total ATP, lactate and phosphate levels. A one h anoxia was chosen for it did not induce a mortality of fed flies. We took care to kill flies in an anoxic state to prevent rapid changes in energy metabolism that could arise as a consequence of reoxygenation. Table 1 show that a 1 h anoxia decreased ATP levels both in anoxia tolerant and anoxia sensitive flies. The net decrease in ATP was larger in anoxia sensitive flies than in anoxia tolerant flies. Thus, anoxia sensitive flies had more ATP than anoxia tolerant flies but they used it more rapidly under anoxic conditions. Table 1 further shows that anoxia induced a production of lactate as expected if anaerobic glycolysis was used to maintain ATP stores. The net production of lactate in response to anoxia was larger in anoxia tolerant flies (8.84 nmoles/fly) than in anoxia sensitive flies (6.63 nmoles/fly). These suggested that anoxia sensitive flies were less efficient to derive energy from anaerobic glycolysis. Finally we observed that anoxia increased tissue inorganic phosphate levels. Inorganic phosphate is a product of the degradation of ATP and other high energy phosphate compounds such as arginine phosphate. The net increase in phosphate content was larger in fed, anoxia sensitive flies (8.28 nmoles/fly) than in anoxia tolerant flies (4.81 nmoles/fly). This result was consistent with the observation that fed flies used their ATP reserves more rapidly than starvation stressed flies.

Taken together these results indicated that feeding flies on a 1S1Y diet induced a major remodelling of their energetic status. Anoxia tolerant flies had less energetic reserves but they used them more sparingly when they were exposed to anoxic conditions.

### Relationship to physical activity and stupor recovery

**Figure 5:**
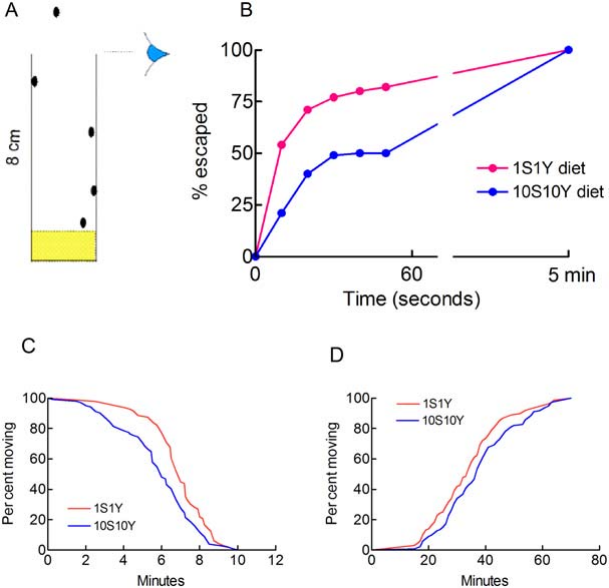
Anoxia tolerance, locomotor activity and stupor recovery. A, B. Feeding flies on a poor diet increases their locomotor activity. A. Schematic representation of the locomotor activity assay (see Material and Methods). B. Escape rates. N = 100 under the two conditions. C. Development of anoxic stupor. Male w^1118^ flies adapted for 2 days to a 1S1Y diet (red, n = 96) or a 10S10Y diet (blue, n = 102) were exposed to anoxia and the time required for each individual fly to fall into stupor was recorded. P = 0.0026 using the log rank test. The time required to decrease oxygen tension to <0.1% was 3 minutes. D. Recovery from anoxic stupor. Male w^1118^ flies adapted for 2 days to a 1S1Y diet (red, n = 136) or a 10S10Y diet (blue, n = 133) were exposed to a 1 h anoxia and reoxygenated. The time required to resume a walking activity was measured. P = 0.04 using the log rank test.

It has previously been shown that starvation stressed flies are more active than fed flies [20]. Figure 5 shows that flies fed on a 1S1Y diet were more active than fed flies.

Maintaining flies at a reduced temperature (18°C) reduces locomotor activity, slows down development and increases the tolerance to chronic hypoxic conditions [9]. We therefore asked whether cold adaptation could produce an anoxia tolerant state and reduce A/R injuries. Flies were adapted for 2 days to a 10S10Y diet at 18°C and exposed to a 3.5 h A/R stress. A/R induced mortality (89%, n = 147) was similar to that of control flies which were maintained at 25°C (94%, n = 199). Thus, a cold stress and the resulting hypoactive state did not protect flies against A/R injuries.

Flies exposed to anoxic conditions fall on their side and stay in a state of stupor. Stupor recovery following reoxygenation has previously been used as a measure of the hypoxic tolerance of flies [11], [21]. We asked whether strong dietary restrictions influenced the development of anoxic stupor and stupor recovery. Flies were fed either on a 1S1Y or a 10S10Y diet for 2 days and exposed to anoxic conditions. Figure 5C shows that flies fed on a poor 1S1Y diet took more time to fall into anoxic stupor than flies adapted to a rich 10S10Y diet. This result was consistent with a better resistance to anoxia. Stupor recovery was not affected by dietary conditions (Figure 5D).

### Pharmacological interventions

**Figure 6:**
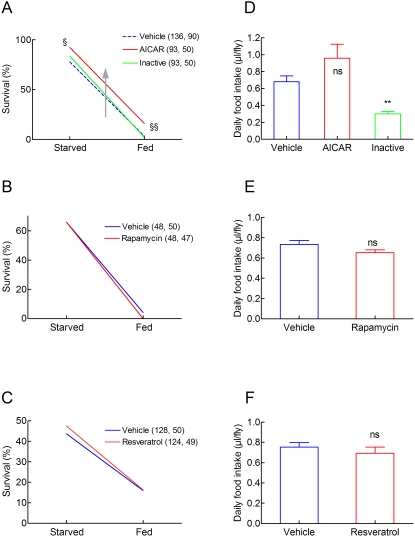
Pharmacological interventions. A. AICAR increases the tolerance of the *w^1118^* flies to A/R stresses in a diet independent manner. Flies were maintained for 2 days on a 1S1Y or a 10S10Y diet supplemented with 100 mM AICAR, 100 mM 5(4)-Aminoimidazole-4(5) carboxamide or vehicle (phosphate buffered saline). They were exposed to a 3.5 h A/R stress, reoxygenated and switched to a 10S10Y diet. Survival was measured after 2 days. Sample sizes (starved, fed) are indicated in parentheses. § p<0.01 as compared to flies treated with the vehicle only and p<0.05 as compared to flies treated with the inactive compound. §§ p<0.01 as compared to the two other conditions. B. Rapamycin does not modify the anoxic tolerance. Flies were maintained for 2 days on a 1S1Y or a 10S10Y diet supplemented with 100 µM rapamycin or vehicle (1% ethanol). They were exposed to a 3.5 h A/R stress, reoxygenated and switched to a 10S10Y diet. Survival was measured after 2 days. Sample sizes (starved, fed) are indicated in parentheses. Mortalities of rapamycin treated flies were not different from those of controls. Low concentrations of ethanol (1%) reduced the survival of starved A/R stressed flies probably by providing calories to the flies and by reducing the strength of dietary restriction. C. Resveratrol does not modify the anoxic tolerance. Flies were maintained for 2 days on a 1S1Y or a 10S10Y diet supplemented with 100 µM resveratrol or vehicle (1% dimethylsulfoxide). They were exposed to a 3.5 h A/R stress, reoxygenated and switched to a 10S10Y diet. Survival was measured after 2 days. Sample sizes (starved, fed) are indicated in parentheses. Mortalities of resveratrol treated flies were not different from those of controls. Low concentrations of dimethylsulfoxide (1%) reduced the survival of starved A/R stressed flies for unknown reasons. D, E, F. Food intakes by flies fed on AICAR or 5(4)-Aminoimidazole-4(5) carboxamide (D), rapamycin (E) and resveratrol (F). Drugs were used at a concentration of 100 mM. Controls were performed using corresponding vehicles. All solutions were supplemented with 5% sucrose. Means±sem are indicated (n = 4–7). ** p<0.01 as compared to vehicle, ns: not statistically different.

We then looked for pharmacological interventions that could modify the tolerance of the flies to A/R stresses and its diet dependence. First, we used AICAR, an agonist of AMP Kinase [22]. AMP kinase is generally quiescent under normal conditions but is activated in response to hormonal signals and stresses sufficient to produce an increase in AMP/ATP ratio, such as hypoglycemia, strenuous exercise, anoxia, and ischemia. Figure 6A shows that AICAR increased the survival of A/R stressed flies both fed and starved. An inactive structural analogue of AICAR (5(4)-Aminoimidazole-4(5) carboxamide) did not. Figures 6B and 6C further show that rapamycin and resveratrol did not modify A/R induced mortality of both fed and dietary restricted flies. Figure 6 (D–F) further indicate that AICAR, rapamycin and resveratrol did not modify the feeding behaviour of the flies as assessed by a capillary feeding assay. The inactive AICAR analogue decreased food intake. These results indicated that AICAR did not increase the anoxic tolerance by a food repellent action.

### A/R injuries in sima loss of function mutants

**Figure 7:**
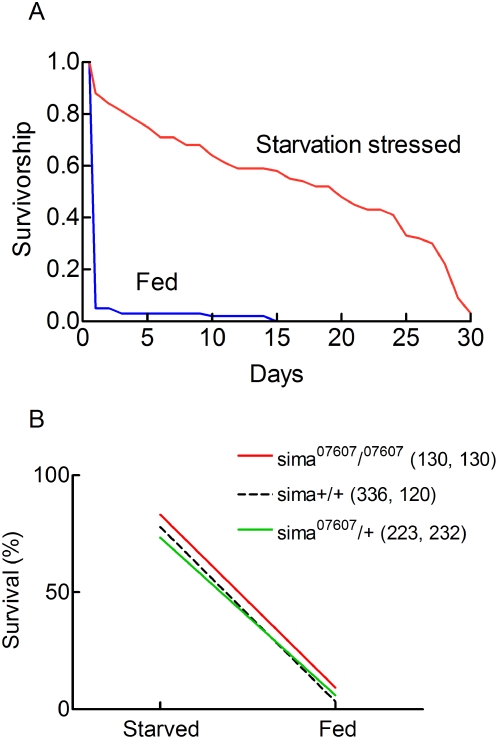
The sensitivity of sima^07607^/^07307^ flies to A/R stresses. A. Sima ^07607^/^07307^ flies were adapted for 2 days to 10S10Y (black) or a 1S1Y (grey) nutrient diet, exposed to a 3.5 h A/R stress, reoxygenated and switched to a 10S10Y diet. Survivorship curves were determined. Mean longevities were 17.1±1.3 days (n = 69) and 1.37±0.3 days (n = 66) for flies adapted to 10S10Y and 1S1Y diet respectively. B. Norms of reaction. Flies of different genotypes were adapted for 2 days to either a 10S10Y or a 1S1Y diet. They were exposed to a 3.5 h A/R stress, reoxygenated and surviving flies were counted after 48 hours. Sample sizes (starved, fed) are indicated in parentheses. Differences between genotypes were not statistically significant.

Hypoxia Inducible factor-1/sima is a master switch in the metabolic and functional adaptation to hypoxic conditions both in mammals [23] and Drosophila [24], [25]. Previous evidence has suggested that the HIF-1/sima signalling pathway is activated in adult anoxic flies [24]. We used sima^07607^/^07607^ loss of function mutants [26] to identify a possible role of HIF-1/sima in A/R injuries. Figure 7A shows that sima^07607^/^07607^ flies behave as control flies. Flies fed on a 10S10Y diet were killed by an A/R stress. Flies adapted to a 1S1Y diet were anoxia resistant. Figure 7B compares the responses of flies of different genotypes. Sima^07607^/^07607^, sima^07607^/+and w^1118^ flies had identical sensitivities to A/R stresses and diet dependences. These results indicated that the HIF-1/sima did not contribute to A/R injuries.

### A/R injuries and insulin signalling

The specific reduction of function mutants of daf-2, an insulin/insulin like growth factor receptor, has previously been reported to promote anoxia resistance in *C. elegans* [27]. The possible role of insulin signalling in the anoxic tolerance of *Drosophila* was evaluated using a series of well characterized mutants of the insulin signalling pathway. Loss of function of chico, the *Drosophila* homolog of mammalian insulin receptor substrate produces dwarf flies that are long lived and stress resistant [28]–[30]. Mutations of the insulin receptor (InRE19 and InREC34) are recessive embryonic or early larval lethal [31], [32]. In the heterozygous state, InRE19 and InREC34 mutants have decreased insulin receptor densities or decreased insulin stimulated tyrosine kinase activity [31], [32].

**Figure 8:**
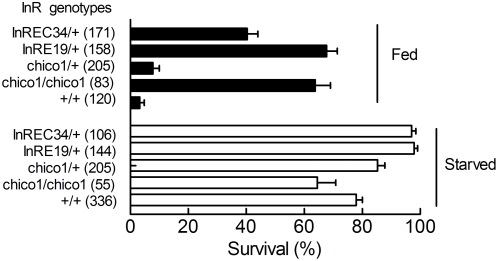
The sensitivity of mutants of the insulin signalling pathway to A/R stresses. Flies of different genotypes were adapted for 2 days to a 10S10Y diet (filled bars) or a 1S1Y diet (open bars), exposed to a 3.5 h A/R stress, reoxygenated and switched to a 10S10Y diet. Surviving flies were counted after 48 hours. Mean±sem are shown. The numbers of flies used are indicated in parentheses.

Flies of different genotypes in a starved or fed state were prepared and their sensitivity to a 3.5 h A/R stress was evaluated. Figure 8 shows that starved flies of the different genotypes were anoxia tolerant. In a fed state, chico1/chico1, InRE19/+and InREC34/+flies were anoxia tolerant. Chico1/+flies were not. It is important to note that identical mortalities rates were observed for A/R stressed chico1/chico1 flies fed on a 10S10Y diet (36%, n = 55) or a 1S1Y diet (35%, n = 83). Thus A/R injuries in chici1/chico1 flies were diet independent.

## Discussion

A/R stresses are deleterious to most mammalian species, including humans. They have immediate consequences and long term consequences that are poorly understood. Here we used the *Drosophila* model to analyse the consequences of A/R stresses on mortality. Results of a demographic analysis indicated that a 3.5 h A/R stress increases the risk of death up to 20 fold. After 10 days, the relative risk of death was still 1.8. This long lasting decrease could be a consequence of a heterogeneous cohort. Sensitive flies die first and leave anoxia tolerant flies. It could also be indicative of the existence of a slow repair process that reduces A/R damage. A deeper anoxia (0.1% O_2_) produces a more transient increase in mortality.

There is considerable evidence that reactive oxygen species contribute to the A/R injuries in mammals [3], [33]. For instance in the mouse, targeted disruption of superoxide dismutase 1 worsens ischemic reperfusion injury [34], and overexpression of superoxide dismutases are cardio- and neuroprotective [35]–[37]. Pharmacological evidence using Euk-8 suggests that in flies too, reactive oxygen species contribute to A/R induced mortality. Further studies using flies with modified ROS scavenger expression are needed to further define the role of ROS in the response of the flies to A/R injuries.

### Different consequences of dietary restriction

This study shows for the first time that the severity of A/R injuries is not a fixed property of the organism. It is possible to reduce A/R injuries by selected dietary manipulations. It is important to note that dietary manipulations have been applied prior to the A/R stress and that all flies have been switched to the same nutrient rich medium after reoxygenation. The possible influence of different dietary conditions after reoxygenation was not analysed.

Dietary restrictions are clearly beneficial to flies. They increase the life span of normoxic flies, reduce fecundity and increase the resistances to starvation and hypoxic stresses [9], [38], [39]. This study shows that strong dietary restrictions protect flies against A/R stresses. Different protocols of dietary restrictions have been used in different laboratories and it is still not clear whether all beneficial actions reported so far are mechanistically related. We previously reported a detailed analysis of the influence of serial food dilutions on the longevity of normoxic flies and of chronically hypoxic flies [13]. This study provides comparable measures of the influence of serial food dilutions on the tolerance to A/R stresses. The results can be summarized as follows: (i) Mild dietary restrictions (2 to 3 fold dilutions of a 10S10Y medium) increase the longevity of normoxic flies and of chronically hypoxic flies. This action is mimicked by a selective yeast restriction. Mild dietary restrictions do not increase the tolerance of the flies to A/R stresses. (ii) Strong dietary restrictions that are close to starvation conditions decrease the longevity of normoxic flies and of chronically hypoxic flies. They promote the tolerance to A/R stresses and their actions cannot be mimicked by a selective yeast restriction. These results suggest that dietary restriction does not induce a general resistance to hypoxic/anoxic stresses. Mild and strong dietary restrictions trigger different responses.

We further report here that cold adaptation, which increases the life span of normoxic flies and of chronically hypoxic flies [9], does not protect flies against A/R stresses. Thus, cold adaptation promotes hypoxic tolerance but not anoxic tolerance. Conversely, loss of function of HIF-1/sima which decreases the hypoxic tolerance of the flies [25] does not modify the sensitivity of adult flies to acute anoxia. Taken together these results indicate that hypoxic and anoxic tolerances can be dissociated and probably involve different mechanisms. A greater tolerance to chronic hypoxia is not accompanied by a greater resistance to A/R stresses.

A/R stresses and chronic hypoxic stresses differ in an important respect. The consequences of anoxic stresses can only be evaluated after reoxygenation of the flies. The consequences of chronic hypoxia can be assessed without reoxygenation for flies remain active. Reoxygenation is well known to induce a massive production of reactive oxygen species which can be as harmful as anoxia [3], [33]. It is important to stress that the responses to anoxia and to reoxygenation are intimately entangled and cannot be dissociated. A decrease in A/R induced mortality can be caused by an increased tolerance to anoxia. It can also result from a decreased sensitivity to the harmful consequences of reoxygenation or from the combined actions of the two mechanisms.

### Diet dependent remodelling of energy metabolism

Feeding flies for 2 days on a poor 1S1Y diet induces a reversible anoxia tolerant state. This state is characterized by lowered ATP, glycogen, triglycerides and protein contents. This study also shows that during anoxia, anoxia tolerant flies produce more lactate, less phosphate and they maintain more stable ATP levels than anoxia sensitive flies. Considering in addition that starving flies produce less CO_2_ [40], these results suggest that poor diets induce a state of metabolic depression. Surprisingly, this state is associated to an increased activity of the flies. It is of interest to note that lower vertebrates, such as frogs and turtles, and hibernating mammals switch to a hypometabolic state under hypoxic conditions [41]. The relationships between these different forms of metabolic depression remain to be determined.

### The mechanisms of anoxic tolerance

Our results clearly indicate that a starvation stress increases the tolerance of flies to A/R stresses. A starvation stress induces a large remodelling of the transcriptome which involves as much as 25% of the *Drosophila* genome [42]–[44]. Anyone of these gene products identified in these screens can contribute to the sensitivity to A/R stresses. Here we used selected pharmacological and genetic tools to delineate some of the mechanisms involved. We show here that rapamycin, resveratrol and sima loss of function do not reduce A/R injuries. Rapamycin and resveratrol have been shown to increase the normoxic longevity of the flies by inhibiting TOR and activating histone deacetylase sir2 respectively [45], [46].

In contrasts pharmacological activation of AMP kinase and defective insulin signalling decreased A/R induced injuries. These results are consistent with the proposed roles of AMP kinase and insulin signalling in nutrient sensing and adaptation [22], [28], [47], [48]. They further agree with two observations (i) ablation of cells making insulin like peptides in *Drosophila* induces a resistance to exogenous oxidative stresses and to starvation conditions [49]. (ii) Worms with defective insulin signalling are anoxia tolerant [27]. Interestingly we further observed that anoxia resistant chico1/chico1 flies are largely diet independent. This suggests that insulin signalling contributed to the diet dependence of A/R injuries.

### Conclusion

Long A/R stresses induce a transient increase in mortality in *Drosophila*. This mortality is highly dependent on dietary conditions prior to the stress. Strong dietary restrictions and starvation conditions protect flies against A/R injuries, probably by inducing a major remodelling of energy metabolism. The results also indicate that mechanistically different responses develop in response to dietary restrictions of different strengths. Finally, this study identifies AMP kinase and the insulin signalling pathway as possible mediators of diet dependent anoxic tolerance in *Drosophila*.

## Materials and Methods

### Flies husbandry

*w^1118^*, Canton S and Oregon R flies were obtained from the Bloomington *Drosophila* Stock Centre at Indiana University. Chico1/CyO flies were kindly provided by Dr. E. Hafen. Chico1/chico1 flies were obtained by crossing chico1/CyO flies and were recognized to their small size and the absence of CyO phenotype. Chico1/CyO flies were used as controls. InRE19/TM2 and InREC34/TM3 flies were kindly provided by Dr. P. Léopold and used in the heterozygous, balanced state. Sima^07607^/TM3 flies were kindly provided by Dr. P. Wappner. Homozygous, loss of function sima^07607^/^07607^ flies were generated by crossing heterozygous sima^07607^/TM3 flies. All flies were reared in 300 ml bottles filled with 30 ml of food medium (8.2% cornmeal, 6.2% sucrose, 1.7% heat inactivated baker's yeast and 1% agar supplemented with 3.75 g/l methyl 4-hydroxybenzoate) and under normoxic conditions. This medium will be referred to as the “standard diet”. Flies were maintained in humidified, temperature controlled chambers at 25°C and 60% relative humidity and under a 12∶12 light∶dark cycle. Aged flies were male w^1118^ flies which had been maintained for 28–31 days on a standard diet.

Defined nutrient media consisted of heat inactivated yeast (Y), and sucrose (S) in variable proportions, 2% agar and 3.75 g/l methyl 4-hydroxybenzoate. Media were labelled according to the following convention. A “10S10Y” medium was a 10% sucrose and 10% yeast nutrient mixture. Starvation conditions (0S0Y) refer to an agar only medium which provided water to the flies. The nutrient media were poured into 30 ml tubes. Tubes were sealed with rubber stoppers to prevent dehydration, stored at 4°C and used within one week. Flies were adapted to defined nutrient media for 2 days before being A/R stressed. All A/R stressed flies were maintained on a 10S10Y diet after reoxygenation.

### Anoxia/Reoxygenation

Experiments were performed using young males w^1118^ flies unless otherwise indicated. Two protocols were used.

#### Protocol 1

Newly emerging males were collected and added to 30 ml vials at a density of 10 flies per vial. The food medium was a 10S10Y medium. After two days of adaptation at 25°C, vials were sealed with natural rubber septa (SubA seal, 22 mm internal diameter, Sigma Chemical Co., St Louis, Mo) equipped with 18G needles [9]. Vials were flushed with 20 volumes of pure N_2_ gas and the needles were removed to seal the atmosphere. Oxygen tension in sealed tubes was measured using a Wittt Oxybaby® oxygen analyser. We determined that it took less than 1 minute to decrease ambient oxygen from 21% to less than 1%. Vials were carefully opened after 2 h (2 h A/R stress) or 3.5 h (3.5 h A/R stress). Oxygen tensions were 0.83±0.02% (n = 133) and 1.31±0.07% (n = 128) after 2 h and 3.5 h of anoxia respectively. Vials were sealed with cotton plugs and further incubated at 25°C. Survival was scored every day. Dead flies were diagnosed by their lack of a sit-up response. This protocol was used in the experiments shown in Figure 1.

#### Protocol 2

Newly emerging males were collected and fed for 2 days on defined food media. Density was ten flies per vial. Vials were sealed with cotton plugs and inserted into the air lock of a “basic glove box” (PLAS LABS, Lansing, MI) maintained at a temperature of 21°C. The transfer chamber was flushed with pure N_2_ gas. After 3 minutes, oxygen tension was reduced to <0.1%. After 10 minutes, all flies had fallen into stupor and the vials were transferred to the main chamber which was equilibrated with pure N_2_. The O_2_ tension in the main chamber was continuously monitored. It was <0.1%. After different periods of anoxia, vials were transferred back to the air lock and then to room atmosphere. Flies were then transferred to a 10S10Y nutrient medium unless otherwise indicated. A/R induced mortality was assessed after 2 days of reoxygenation. This protocol allows deeper anoxia than protocol 1 (0.1% O_2_ vs. 1% O_2_).

### Sensitivity to drugs

All drugs were obtained from the Sigma Chemical Co (St Louis, Mo). AICAR (5-aminoimidazole-4-carboxamide-1-beta-D-ribosefuranoside and its inactive homologue (5(4)-aminoimidazole-4(5) carboxamide) were dissolved into phosphate buffered saline (pH 7.5). Rapamycin was dissolved into 1% ethanol. Resveratrol was dissolved into 1% dimethylsulfoxide. 250 µl aliquots were layered on the top of freshly prepared 10S10Y or 1S1Y media and allowed to adsorb for at least 24 hours at room temperature. Controls were performed using the vehicle only (phosphate buffered saline, 1% ethanol or 1% dimethylsulfoxide). All flies were switched to a drug free, 10S10Y diet after reoxygenation.

The influence of drugs on feeding was assessed using the capillary feeding assay [50]. A 75 µl capillary was filled with a 5% sucrose solution and drugs (or vehicle). One day old male flies in groups of 6 were exposed to the different solutions for 48 hours. Intakes were determined using callipers and daily consumptions were computed and corrected for evaporation. Mean daily intakes were determined using 4–7 groups of 6 flies.

### Glycogen, triglycerides and protein contents

Flies in groups of 20 were homogenized in 200 µl of 25 mM Hepes buffer (pH 7.5) supplemented with 5 mM of 3-[(3-cholamidopropyl)dimethylammonio]-1-propanesulfonate. The homogenate was centrifuged for 30 minutes at 4°C and 13,200 rpm. Glycogen was assessed as amylase released glucose using the Sigma starch assay kit. Triglycerides were assayed using the Sigma TG determination kit (TR0100). Total proteins were determined according to Bradford and using reagents from Biorad.

### ATP assay

Flies in their normoxic or anoxic atmosphere were frozen in liquid nitrogen. Flies in groups of 4 were homogenised in 100 µl of a 5% perchloric acid solution. The extract was centrifuged for 15 minutes at 4°C and 13,200 rpm. The supernatant was neutralised with a solution of KOH 0.25 M/KH_2_PO_4_ 1 M (4/1) at 4°C. The perchlorate precipitate was removed by centrifugation and ATP was measured in the supernatant using the Sigma ATP Luminescent AC kit.

### Lactate and phosphate assays

Lactate and inorganic phosphate contents were determined by ion exchange chromatography using an IonPac AS11 column (Dionex Corporation, Sunnyvale, Ca). Flies in groups of 34 were homogenized in 250 µl of distilled water. The homogenate was centrifuged for 15 minutes at 4°C and 13,200 rpm. Aliquots of the supernatant (10 µl) were diluted 1∶12 into distilled water and 50 µl samples were loaded onto the ion exchange column. The column was eluted at a rate of 1 ml/minute with a linear gradient of 12–35 mM KOH. Areas of eluted lactate and phosphate peaks were calibrated using various dilutions of home made standard solution of 10 mM sodium lactate and sodium phosphate in distilled water.

### Testing the influence of Euk-8 on A/R responses

Euk-8 (manganese N,N′-bis(salicylidene)ethylenediamine chloride) was purchased from Calbiochem (Merck, Darmstadt, Germany). It was used under the conditions defined by Magwere et al. [12]. Euk-8 was added to a 10S10Y food mixture to obtain a final concentration of 1 mM. We took care to cool the food mixture to 50°C before addition of Euk-8. One day old flies were fed for 2 days on a Euk-8 supplemented diet. Half of the flies were exposed to a 3.5 h anoxia and reoxygenated using protocol 1. The remaining flies were maintained at atmospheric oxygen. A/R induced mortality was determined after 48 hours. The possibility that Euk8 acted as a food repellent and produced a starved state was considered. The mean longevity of flies fed on a 10S10Y diet supplemented with 1 mM Euk8 was 24.0±0.6 days (n = 90). It was less than the mean longevity of flies maintained on a drug free diet (30.9±0.7 days, n = 109), thus confirming the observations of Magwere et al., [12]. The mean longevity of flies maintained on a 1S1Y diet (10.5±0.4 days, n = 196) or of starving flies maintained on an agar only medium (4.1±0.1 days, n = 280) were much shorter. These results indicated that Euk8 did not induce a starvation like state.

### Locomotor activity

We used a startle-induced excitability assay described previously [51]. Briefly, 5–10 flies were allowed to adapt either to a 10S10Y or a 1S1Y nutrient mixture. After 2 days, flies were tapped 5 times to the bottom of the tube to startle them. The cotton plug was removed and the tubes were left in an upright position. Flies rapidly recovered from startle and climbed up the tubes. The numbers of flies which escaped from the tube (i.e. that had moved a distance of 8 cm) were counted every 10 s.

### Statistical analysis

Survival data were compared using the log rank test (GraphPad Prism 4) and the Cox regression model (StatPlus, AnalysSoft). Median survival, mean survival, maximum survival and the relative risk of death were determined. Maximum survival was defined as the median longevity of the final surviving 10%. A/R induced mortalities were compared using the z-test. Results of biochemical experiments are expressed as means±sem. The numbers of independent experiments performed are indicated. Means were compared using t tests. P values <0.05 were considered as statistically significant.
